# Subjective Social Mobility among Migrant Children in China

**DOI:** 10.3390/ijerph19095685

**Published:** 2022-05-07

**Authors:** Wei Lu, Nian Liu, Juan Chen

**Affiliations:** 1 Department of Social Work, School of Sociology and Anthropology, Xiamen University, Xiamen 361005, China; vivia1009@xmu.edu.cn; 2 Department of Sociology, School of Public Administration, Guangzhou University, Guangzhou 510006, China; 3 Department of Applied Social Sciences, The Hong Kong Polytechnic University, Hong Kong SAR, China; juan.chen@polyu.edu.hk

**Keywords:** migrant children, subjective social mobility, social ecology

## Abstract

Little scholarly attention has been paid to the relationship between children’s subjective social mobility and their “social ecology”. Children’s subjective social mobility is about how they perceive their future social position compared to their parents’. Social ecology refers to the influential multi-layered surrounding factors, including family, school, and community. We analyzed data from structured questionnaires completed by 2221 migrant children (1296 boys and 925 girls, with a mean age of 11.7 years) from three private schools in Guangzhou and Foshan, China. The findings indicate that participants anticipated a significant improvement in their future social status. Of the factors influencing this belief, community integration has the most significant impact (*TE* = 0.246), followed by school integration (*TE* = 0.220) and family socioeconomic status (*TE* = 0.053). We also found that children’s self-concept plays a role in their perceptions of social mobility. Based on the study results, we propose recommendations to provide migrant children additional protection and enhance their living environment.

## 1. Introduction

“Subjective social mobility” refers to individuals’ view of the likelihood of improving their social class [1]. Scholars argue that children’s subjective social mobility reflects their positive expectations and sense of control regarding the future; these, in turn, will boost their psychological resilience and reduce the pressure of financial difficulties [2]. Positive perceptions of social mobility can also promote attitudes, leading to socioeconomic success (eagerness to improve academic performance, for example), particularly among adolescents with lower socioeconomic status [3,4]. Empirical studies have demonstrated that children’s beliefs about achieving a higher future social status (i.e., higher than their parents’) encourage academic motivation and resilience in the face of adversity [3,5]. However, little attention has been paid to determining the factors that enhance subjective social mobility and how they promote children’s development. Addressing this research gap will allow us to help children reach their potential.

Family migration has been a major trend in China’s urbanization process [6]. According to the Seventh National Population Census [7], China’s migrant population was 376 million in 2020. At least one-third of these—130–150 million—were children, accounting for half of China’s total population of 0–17 year-olds. Of these, half (50.76%) were of compulsory education age. Although migrant children’s access to public services and welfare benefits has increased in recent years, difficulty in enrolling in local schooling, high costs (including living costs and education expenses), and inadequate childcare remain the three main problems regarding children’s compulsory education reported by 20–30% of migrant parents [7].

Studies have focused on the relationship between migration and children’s life satisfaction [8,9]; however, scant attention has been paid to the environmental variables (e.g., socio-economic status, school, and community integration) and personal variables (e.g., self-concept and class identification) that may affect migrant-related disparities in children’s subjective social mobility. Therefore, the first aim of our study was to examine how children’s subjective social mobility relates to what Bronfenbrenner termed “social ecosystem”. Our second aim was to determine whether children’s self-concept moderates the relationship between subjective social mobility and their social ecosystem.

### 1.1. Migrant Children’s Self-Concept and Their Social Ecosystem

According to Bronfenbrenner [10], the social ecology of human development comprises several layers. At the center is the individual- or self-system, influenced by personal characteristics and genetic factors. Piers and Harris [11] use the term “self-concept” to refer to children’s feelings, attitudes, and evaluations of their own behaviors, abilities, and values. Children’s self-concept reflects their understanding of their own environment and social status [11]. 

Bronfenbrenner identified the layer surrounding the self-system—the first layer of social influence—as the microsystem. His later work focused on the impact of children’s social experiences on their individual psychology [12]. Children are affected by the dynamics of their family’s parenting style [13], classroom relationships with their teachers [14], and their peers’ norms and behaviors [15], among many other interactions [6]. These influences represent the child’s closest physical and psychological attachments.

The next layer of the ecosystem identified by Bronfenbrenner [10] is the exosystem, which contains “one or more settings that do not involve the developing person as an active participant, but in which events occur that affect, or are affected by, what happens in the setting containing the developing person.” Their parents’ workplace or the labor market, for example, are part of an exosystem in which children do not participate. However, when a parent loses their job, their ability to raise their children is affected, in turn increasing the possibility of their children experiencing behavioral and emotional difficulties [16].

Migrant children in China have been variously portrayed in the literature. Some studies argue that many migrant children have a positive view of life [17,18] and optimistically assume that their future circumstances will improve [19]. Other studies show that they suffer from a range of psychological problems, such as anxiety, loneliness, poor self-image, and behavioral issues [20].

Scholars have discussed the impact of parenting styles, teachers, social culture, and peer pressure on the development of migrant children’s self-concept [21,22]. Migrant children are more affected by teachers, teaching methods, learning facilities, the availability of resources, and the quality of teacher–student relationships than other children [23]. They tend to have a more robust self-concept and stronger sense of belonging among other migrant peers [24]. Due to their limited social networks [25] and limited acceptance in the community [26], migrants often experience difficulty becoming part of the neighborhood and accessing community resources. Because of this loss of social capital, migration often negatively impacts children’s academic performance [25].

### 1.2. Research Framework and Hypotheses

**Figure 1:**
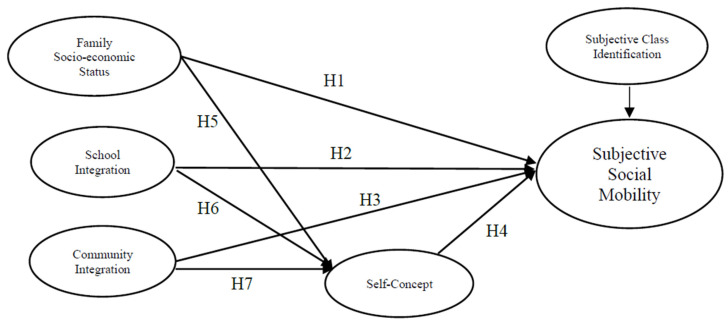
Theoretical framework of migrant children’s subjective social mobility.

Based on the findings outlined above, we hypothesized that migrant children’s social ecosystem will significantly affect their subjective social mobility. As shown in Figure 1, their ecosystem includes families’ socioeconomic status (H1), their degree of school integration (H2), and their degree of community integration (H3). We further hypothesized that children’s self-concept can influence the effects of their ecosystem (H4, H5, H6, H7).

**Hypothesis** **1.** *Family socioeconomic status affects children’s subjective social mobility. The lower the family’s socioeconomic status, the lower their subjective social mobility*.

Migrant children mostly become aware of their family’s lower socioeconomic status after moving to cities [20]. They experience negative emotions, including anxiety, during the adjustment stage [27], which will negatively impact their subjective social mobility.

**Hypothesis** **2.** *The degree to which migrant children become integrated into their school environment affects their subjective social mobility. The better the school integration, the higher the subjective social mobility*.

Studies have documented the psychological effects of peer relationships [24] and teacher–student relationships [23] on migrant children. Therefore, we assumed that the degree of school integration has a significant impact on the subjective social mobility of migrant children.

**Hypothesis** **3.** *The degree to which migrant children become integrated into their community affects their subjective social mobility. The better the community integration, the higher the subjective social mobility*.

Huang et al. [5] note that we should not underestimate the degree to which migrant children are proactive in their new living environment. Migrant children’s initiative is encouraged when they feel accepted and integrated into the community environment, resulting in a higher commitment to class mobility.

As ecosystem activities occur in children’s external world, their influence on children’s subjective social mobility may not always be direct and straightforward. To better understand the mechanism underlying the relationship between children’s ecosystems and their subjective social mobility, we should explore the potential mediators of this relationship. Studies have confirmed that a negative environment affects children’s motivation and self-concept [28,29]. Research has further emphasized the important role of children’s self-concept in forming aspirations [30,31]. Therefore, subjective social mobility is likely influenced by an individual’s self-concept [32]. As a result, it is further assumed that migrant children’s self-concept mediates the impact of the ecosystem on their subjective social mobility. Figure 1 outlines the specific research hypotheses.

**Hypothesis** **4.** *Migrant children’s self-concept affects their subjective social mobility. The better the self-concept, the higher the subjective social mobility*.

**Hypothesis** **5.** *Migrant children’s self-concept mediates the influence of family socioeconomic status on their subjective social mobility*.

**Hypothesis** **6.** *Migrant children’s self-concept mediates the influence of school integration on their subjective social mobility*.

**Hypothesis** **7.** *Migrant children’s self-concept mediates the influence of community integration on their subjective social mobility*.

People’s class identification also affects subjective social mobility [33,34] because their values, happiness, and political attitudes are related to their perceptions of their position on the social ladder [35]. As Figure 1 shows, migrant children’s class identification is controlled in the model. In addition, migrant children may present different path coefficients and mediating effects, according to the gender and educational level. In the analysis, therefore, we also accounted for children’s gender and educational level.

## 2. Methods

### 2.1. Data Collection and the Study Sample

Data were collected in Guangzhou and Foshan from May to June 2019. To qualify for inclusion in the study, participants had to be younger than 18 and had to attend primary school or junior high school in Guangzhou or Foshan. In addition, neither they nor their parents could have local *hukou* (household registration). 

Guangzhou and Foshan are located in the Pearl River Delta—a cluster of cities in China with many factories located in various industrial parks. Migrant workers residing near their workplace comprise a high proportion of the population. Because migrant workers usually do not have local *hukou*, it is very difficult for their children to attend public schools; they usually attend private schools established specifically for them. Our research was carried out in three industrial parks: Xinya Industrial Park (Huadu District, Guangzhou), Light Textile Industrial Park (Haizhu District, Guangzhou), and New Hardware Industrial City (Chancheng District, Foshan), which are home to many industries, including textiles, machinery, commerce and trade, catering, and service. They are typical of areas in Pearl River Delta, with their high concentrations of migrant workers.

From each industrial park, we selected one school that met our four criteria: it was private; the student body comprised children of migrant workers in the industrial park or nearby factories; it offered a nine-year program of compulsory education from primary to junior high school; and it agreed to participate and allowed us to conduct surveys on campus.

We employed cluster sampling to select classes from the three participating schools. Taking into account students’ literacy and comprehension levels, we targeted 93 classes from the third to ninth grade. Because of the limited research funds and human resources, we randomly selected 46 classes using a systematic sampling method. First, we sorted all 93 classes by a class serial number. Then, 46 classes were selected from the ranked class list in intervals of 2. Questionnaires were distributed to all students in the 46 selected classes. A total of 2336 students volunteered to participate in the survey. The questionnaires were distributed during classroom time and completed anonymously. Each class was overseen by three members of the research team. 

Of the 2336 participants, 34 did not complete the questionnaire, 28 submitted questionnaires that were excluded because of missing data, and 53 were excluded because they had local *hukou*, leaving a sample of 2221 for analysis that included 1296 boys (58.4%) and 925 girls (41.6%). Most (1360 or 61.2%) were primary school students in grades three to six; the remaining 861 (38.8%) were junior high school students in grades seven to nine.

According to the Population Change Survey of Guangdong Province in 2013 [36], there were 4.47 million migrants aged 0–17 in Guangdong, of whom 30.58% were aged 8–14 (in general corresponding to grades three to nine). Among migrant children aged 8–14, those aged 8–11 accounted for 63.2%, and those aged 12–14 accounted for 36.8%. Furthermore, 57.4% were male and 42.6% female. The age and gender distributions of the study sample are similar to those of the target population (see Table 1), indicating the representativeness of the study sample.

### 2.2. Measurements

#### 2.2.1. Subjective Class Identification and Social Mobility

Kraus et al. [37] use the term “subjective class identification” to refer to individuals’ perception of their social class status, and, as we have noted, “subjective social mobility” refers to their goal of attaining a higher social class. To make these concepts accessible to children, we asked them to visualize social classes as different floors of a building to indicate which floor represented their current social status (subjective class identification) and which represented their social status expectations (subjective social mobility). This analogy has been used previously in studies of the rural-to-urban migrant population in China with proven reliability and effectiveness [8]. The specific question was framed as follows:

“Imagine the world is a 10-story building, and all people live in it. A higher floor means more resources and higher achievements and prestige. For example, people living on the ninth floor have more resources and have achieved more than those living on the sixth floor. Everyone’s ability to climb to higher floors is based on their own efforts to improve themselves. You think you are living on the ____ floor now. When you grow up and work, you will live on the ____ floor. (Please fill in a number from 1 to 10 in each space.)”.

Data regarding which floors participants thought they will live on were directly assigned to measure subjective social mobility as the dependent variable. Subjective class identification was used as a control variable to eliminate the difference with the current stratum. In the event that the current class status of the sample was the same, the impact of other factors on subjective social mobility was revealed.

#### 2.2.2. Family Socioeconomic Status

A family’s socioeconomic status is determined by factors such as family income, living conditions, parents’ level of education, and occupational status [6,38]. The measures used in the study for family monthly income were as follows: 1 = CNY 4500 and below, 2 = CNY 4501–7000, 3 = CNY 7001–9500, and 4 = CNY 9501 and above. In 2019, the official criteria for low-income families in Guangzhou and Foshan were CNY 1515 and CNY 1470 per month per person, respectively. Considering that a family generally consists of three people, families with a monthly income less than CNY 4500 could be regarded as low-income families. The China Household Wealth Survey Report 2019 [39] indicates that families with a monthly income greater than CNY 9000–10,000 could be viewed as wealthy families.

Living conditions were coded according to three levels: poor (1 = lodging or shared rental), average (2 = independent rental), and good (3 = home ownership). Parents’ education was coded: 1 = junior high school or below, 2 = vocational or technical school, 3 = senior high school, or 4 = college or above. Migrant workers are clustered in labor-intensive industries; thus, parental occupation was not included due to high homogeneity. 

When determining the measures for family socioeconomic status, we took into account that it is a latent variable. Family income, living conditions, and parents’ education level each represent partial characteristics of family status and complement one another. Because they are indispensable manifestations composing the family socioeconomic status factor, they are formative indicators. In the structural equation, causality was directed from the measurement variables (family income, living conditions, and education level) to the latent variable (family socioeconomic status). We processed ordinal variables as interval variables. Based on the children’s understanding of their family’s original circumstances, family income, living conditions, and parents’ education levels were measured by ordinal multi-categorical variables to obtain more accurate data information and improve the completion rate. An interval of CNY 2500 was used for the family income item. Although it was impossible to evaluate family living conditions and parents’ education level with fixed intervals, they were expressed in the ranges of “poor to low” to “good to high”. These parameters were taken as interval variables in the subsequent statistical analysis. 

#### 2.2.3. School Integration

School integration depends on various factors [40,41]. Migrant children’s school integration was determined according to four measures: emotional attachment to school, school performance, classmate relationships, and teacher–student relationship. Participants’ responses to various statements were recorded on a five-point Likert scale. To determine their emotional attachment to school, they were asked if they “liked the school”, “were proud of the school”, “were satisfied with the school”, and “considered school education helpful”. For school performance, they were asked if they “raised their hand to speak in class” and “observed school discipline”. To determine their classmate relationships, they were asked if they “proactively interacted with classmates”, “accepted help from classmates”, “had conflicts with classmates”, and “were bullied by classmates”. The teacher–student relationship was measured by their ratings of the teacher’s “frequency of communication”, “care level”, “praise and encouragement”, and “criticism”. Negative statements were reversely recoded. The higher the score, the better the school integration.

#### 2.2.4. Community Integration

We defined community integration as the closeness between migrant children and their current residential communities. Our five measures focused on three main aspects: attitudes toward the residential communities (“like the residential community”, “like the community residents”), interactions with community residents (“seek help from community residents when encountering a difficulty”), and perceived attitudes of community residents (“am liked by community residents”, “am discriminated against by community residents”). All answers were recorded on a 5-point Likert scale. Negative statements were reversed for corresponding statistical analysis.

#### 2.2.5. Self-Concept

The Piers–Harris Children’s Self-Concept Scale [11] was included in the questionnaire. Su and Liu [42] translated the scale into Chinese, evaluated its reliability and validity, and revised it accordingly. The Self-Concept Scale (Chinese version), which relies on children’s self-evaluations, consists of 80 yes-or-no items covering six areas: behavior, intellectual and school status, physical appearance and attributes, anxiety, popularity, and happiness and satisfaction [43]. The higher the score, the better the children’s self-concept.

### 2.3. Statistical Analysis

Partial least squares (PLS) regression, combined with principal component analysis and multiple regression analysis, was used to estimate the model parameters to maximize the model’s predictive ability. PLS regression is a variance-based structural equation model highly regarded by researchers for use in various fields and applications [44]. PLS regression has unique characteristics that are superior to covariance-based structural equation modeling [45]: the data to be analyzed are not required to have a multivariate normal distribution; it is capable of handling multi-dimensional models with complex structures and processing reflective indicators and formative indicators simultaneously; it is less affected by multicollinearity; it is especially suitable for model prediction; and it provides the best explanations for endogenous variables [46]. The preferred application of PLS regression is with small samples, but it can also be applied to a large sample. In the case of a large sample, PLS presents good consistency in parameter estimates [47]. 

SmartPLS 3.0 software statistical analysis was employed for several reasons. First, the structural model we constructed was relatively complex, covering second-order dimensions, mediating variables, and group comparisons. Second, family socioeconomic status—a formative factor in our study—could be more conveniently processed with SmartPLS software. Third, SmartPLS can calculate and present statistics that explain the maximum relationship between subjective social mobility and the impact of migrant children’s environment and self-concept. 

The reliability and validity of each dimension of factors were verified first. Table 2 shows that the factor loadings for emotional attachment to school, teacher–student relationship, classmate interaction, and school performance exceeded 0.70, and the composition reliabilities (CR) were 0.903, 0.873, 0.874, and 0.882, respectively; the average variance extracted (AVE) was 0.699, 0.633, 0.634, and 0.789, respectively, which are all higher than 0.50, suggesting good discriminative validity. The factor loading for school integration was 0.722–0.823 (CR = 0.842, AVE = 0.577). School integration showed good composite reliability and discriminative validity. The factor loading of the community integration dimension was 0.691–0.791 (CR = 0.858, AVE = 0.548), and the factor loading for self-concept was 0.712–0.866 (CR = 0.908, AVE = 0.624); therefore, both of these factors had good composite reliability and discriminative validity. Subjective class identification and subjective social mobility were single-measurement indicator dimensions. Family socioeconomic status was a formative indicator dimension, with item weights ranging from 0.200 to 0.578 (*p* < 0.05), the variance inflation factor (VIF) ranging from 1.026 to 1.596, and insignificant collinearity, indicating that the index construction was appropriate.

Table 3 shows the results of testing the discriminative validities of the model factors. Family socioeconomic status is the formative factor. The Pearson correlation coefficients for other factors were less than 0.70, showing good discriminative validity. According to the Fornell–Larker criterion [48], when the AVE square root value of a single factor is greater than the Pearson correlation coefficients (*r*) of the factor, and other factors and the heterotrait–monotrait ratio values are less than 0.85, there is good interdimensional discriminative validity. Thus, all factors in the research model were rated highly for reliability and validity.

Having established the reliability and validity of the measurement model, the structural model was statistically analyzed. First, descriptive analysis of the mean, standard deviation, and median of each factor in the structural model was carried out to determine participants’ current circumstances and the distribution of subjective social mobility. To test the research hypotheses, we then calculated the path coefficients between the various factors of the structural model after controlling for subjective class identification. Then, participants’ self-concept was included as a mediating variable. The mediating effects of family socioeconomic status, school integration, and community integration on subjective social mobility and their total effects were tested separately, and the influence, performance, and importance of each factor were calculated. Finally, we distinguished between boys and girls and between primary and secondary school groups to make intergroup comparisons that would show the moderating effects of gender and education.

## 3. Results

### 3.1. Socio-Demographic Characteristics of Migrant Children

Descriptive statistical analysis was conducted on the factor averages of each dimension of the structural model. As Table 4 shows, participants believed that they were currently members of the middle or lower classes, but hoped to become members of the middle or upper-middle classes. On a scale of 1 to 10, their average subjective class identification factor was 3.869, and their average subjective social mobility factor was 6.719. A paired *t*-test conducted between subjective social mobility and subjective class identification (*M* difference = 2.849, *t* = 48.631, *df* = 2220, *p* < 0.001) indicated that participants had a clear determination to progress to a higher class and generally hoped that they would improve their future social class.

Table 4 also shows that the children’s parents had low levels of education, as the education of at least half ended in junior high school or earlier (fathers: *M* = 1.892, *SD* = 1.081, *Mdn* = 1.000; mothers: *M* = 1.764, *SD* = 1.025, *Mdn* = 1.000). The mean and median total monthly family income scores were both close to 2, corresponding to the income range of CNY 4500–7000. According to the 2018 Statistical Yearbook of Guangzhou City [49] and the 2018 Statistical Yearbook of Foshan City [50], the average monthly salaries of Guangzhou and Foshan employees were CNY 9320 and CNY 6691, respectively, at the end of 2018. Given their much lower family incomes, it is not surprising that only 18% of participants’ families had bought a house locally. Most (73.4%) lived in rental accommodation and 8.6% lived in relatives’ or friends’ homes or shared rental accommodation with others. Overall, migrant children’s socioeconomic status was low. 

Participants showed favorable levels of school integration. The average school integration factor was 3.905. Among the first-order factors of school integration, the teacher–student relationship had the most impact (*M* = 4.226) and school performance had the least (*M* = 3.719). The average community integration factor was 3.779, and the average self-concept factor was 0.681—both representing fairly acceptable levels.

### 3.2. Path Coefficient, Mediating Effects, and Model Validity

The structural model fit analysis shows SRMR = 0.084, rms_Theta = 0.121, and NFI = 0.902, meeting the requirements [51] and confirming the model’s acceptability. The bootstrap method was used to repeat sampling 5000 times to estimate the path coefficients among the factors in the structural model. Table 5 shows that the path coefficient (standardized) of participants’ subjective class identification on subjective social mobility was β = 0.245 (*p* < 0.001). The path coefficient of subjective class identification on self-concept was β = 0.059 (*p* < 0.001), indicating that subjective class identification indeed played a controlling role. Self-concept has a significant positive effect on subjective social mobility, with estimated path coefficient β = 0.125 (*p* < 0.001).

Migrant children’s family socioeconomic status, school integration, and community integration all have a significant impact on their subjective social mobility. The direct effect path coefficients were 0.046, 0.165, and 0.213, respectively, and the *p*-values were all less than 0.05. Of the three, community integration had the greatest impact on migrant children’s subjective social mobility, followed by school integration and family socioeconomic status.

Self-concept entered in the structural equation model as a mediating variable to estimate its effect on the path of the child’s ecosystem, affecting subjective social mobility (see Table 6). After repeating the sampling 5000 times with the bootstrap method, the estimated mediating effect of self-concept on family socioeconomic status was 0.008 (*p* < 0.01). Under 95% confidence interval conditions, neither percentile nor bias-corrected results contained 0, suggesting a significant mediating effect. Regarding the effects of school integration and community integration on the children’s subjective social mobility, the mediating effects of self-concept were 0.005 (*p* < 0.001) and 0.033 (*p* < 0.001), respectively. Again, neither percentile nor bias-corrected results contained 0, suggesting significant mediating effects. The findings reported in Table 6, together with those in Table 5, show that the path coefficients of the direct effects of family socioeconomic status, school integration, and community integration were all significant, and self-concept played a mediating role in the relationship between migrant children’s ecosystem and their subjective social mobility. A comparison of the total effect point estimates, taking into account the mediating effect of self-concept, showed that community integration (*TE* = 0.246) had the greatest impact on migrant children’s subjective social mobility, followed by school integration (*TE* = 0.220) and family socioeconomic status (*TE* = 0.053).

Table 7 presents the estimated validity of the structural model and the influence, performance, and importance of the factors in the structural model. Subjective social mobility was an endogenous variable; for this factor, the model’s explanatory ability was *R*^2^ = 0.277, suggesting a medium to high explanatory ability [52]. Using blindfolding matrix clustering technology, latent variables were used to predict observed variables, and Stone–Geisser’s *Q*^2^ value was calculated to evaluate model quality and predictive correlation [53]. Table 7 shows that the structural model had a *Q*^2^ of 0.271, indicating good predictive correlation of the structural model. Although participants’ subjective class identification was a control variable, it had the greatest influence on subjective social mobility (*ƒ*^2^ = 0.080). Again, we see that of the variables representing the child’s ecosystem, community integration (*ƒ*^2^ = 0.043) had a higher impact on subjective social mobility than school integration (*ƒ*^2^ = 0.022) or family socioeconomic status (*ƒ*^2^ = 0.003). In the diagram of the structural model paths, subjective social mobility was the final endogenous variable for the importance–performance map analysis of each variable. Performance was measured using the average latent variable score (0–100 points): the higher the score, the better the performance of the latent variable in the structural model. Importance, expressed in nonstandardized values, shows the absolute total effect of each variable on subjective social mobility. The larger the value, the more important the variable in the model [51]. Table 7 shows that both community integration and school integration had high levels of performance and importance, whereas family socioeconomic status had relatively low performance and importance in terms of subjective social mobility.

### 3.3. Testing the Moderating Effects of Gender and Education

To determine whether gender and education were moderating variables, we divided participants into groups according to gender and level of education (primary or secondary school) and used a multi-group permutation algorithm to make comparisons. First, the factor loading of each measurement variable, factor mean, and factor variance in the structural model were compared to establish the invariance of the factor measurement and ensure that any model difference was not caused by the groups’ different perceptions of the measurement model. After random repeated sampling (without replacement) 5000 times, the factor loading and weight difference of each measurement variable were calculated for boys and girls, respectively. The 5000 values were arranged from small to large to observe whether the interval formed by the 2.5% to 97.5% percentile difference contained 0 (Table 8). The factor loadings or weights of all measurement variables in both gender groups were identical, as were the means and variance of each factor. Similarly, the primary and secondary school groups were identical in factor loadings or weights, means, and variances. 

Permutation comparison was conducted on the path coefficients and mediating effects to determine whether the structural model showed differences between genders and education levels. Table 9 shows that the difference between the original path coefficients of the two genders was relatively small; after repeated sampling 5000 times, the range between the 2.5% and 97.5% percentiles of the permutated average differences contained 0, so gender showed no significant effect on path coefficients. When the mediating effect of self-concept was applied to the two genders, the interval between the 2.5% and 97.5% percentiles indicated that self-concept had the same mediating effect between environmental factors and subjective social mobility for both genders. Similarly, the primary and secondary school groups showed no significant differences in the path coefficients and self-concept mediating effects.

## 4. Discussion and Conclusions

In our analysis of the data provided by 2221 migrant children in Guangzhou and Foshan, family socioeconomic status (Hypothesis 1), school integration (Hypothesis 2), and community integration (Hypothesis 3) all showed significant positive impacts on subjective social mobility, confirming all three hypotheses. We also found that the higher the children’s self-concept, the higher their subjective social mobility, providing support for Hypothesis 4. Self-concept had a significant mediating effect on the relationship between environmental factors and subjective social mobility (supporting Hypotheses 5–7). The model has a certain explanatory power and good predictive correlation for the differences in subjective social mobility. Community integration showed the greatest impact on subjective social mobility, followed by school integration, while family socioeconomic status had the least impact. Differences in gender and level of education did not affect path coefficients or self-concept mediating effects. 

### 4.1. Migrant Children’s Self-Concept as a Protective Factor

The significant impacts from the first three confirmed hypotheses support discussions of how children’s environments, from the child’s perspective, influence their subjective social mobility, but in a more systematic logic. It is believed that a negative environment might easily lead to negative psychological effects and subjective judgments of their social status, which would lead to lower behavioral motivation [28,54,55,56]. The economic status of migrant families has been structurally solidified by national social and economic status, which is difficult to change in the short term. Migrant parents have very few resources for class mobility, and they cannot make the next generation see the effect of any class change in the short term. As a result, migrant children cannot have a broader social vision, and it is indeed difficult to break through the existing ceiling in terms of cognition. In schools and communities with high migrant population homogeneity, the higher the acceptance level of migrant children from people with the same background in the school and community networks, the more confidence and courage they can gain to fight for the future. Conversely, if people around them, especially fellow migrants, fail to provide sufficient support, migrant children will feel more alienated from the city and lose their mobility.

Recommendations based on the above conclusions involve adjustments in the environment. Our research also shows that migrant children’s self-concept plays a key role in their subjective social mobility. More importantly, although they evaluated the status quo relatively negatively, their subjective social mobility remained positive. Various environmental factors appear to affect them at different levels and intensities. The findings indicate that a positive self-concept plays a protective role. 

Previous research suggests that children’s self-concept is closely related to their growth cycle—by secondary school, self-concept shows a downward trend due to growing academic pressure and more complicated relationships with peers [57]. Nevertheless, our study shows that the mediating effect of self-concept did not differ by education stages, which might be explained by the formation mechanism of migrant children’s self-concept in cities. As migrant children grow older and gain more experience, they will have a deeper understanding of their parents’ situation and family conditions and adjust their self-concept. Given that their schools and communities are characterized by high concentrations of migrants, their sense of relative deprivation is low, and school and community integration is relatively high due to homogeneous relationships. Thus, although migrant children live in a non-ideal ecosystem, it still positively influences their subjective social mobility through the mediating effect of a favorable self-concept. Nonetheless, when migrant children are separated from highly homogeneous learning and living groups, their self-concept needs special attention and protection.

### 4.2. Reconstructing the Service System for Migrant Children

It is generally assumed that migrant children’s subjective expectations are shaped first by their family, then by their school, and, finally, by their community. However, our findings suggest the reverse: family socioeconomic status has the least effect, school integration follows, and community integration is most influential. This surprising finding can be attributed to two characteristics of migrant children. 

The first is their sense of involvement. For migrant children, the family’s socioeconomic status is a given entity, established without their direct involvement. Schools and communities, on the other hand, require their participation. If the children feel involved and actively influential in these areas, they become optimistic about their future. 

Second, migrant children realize that there are more social resources in cities than in their rural birthplace. They can become part of a large social network in their highly homogenous communities [58], which increases their social confidence. As a result, they have higher expectations for future class mobility and are motivated to achieve it.

Therefore, the social service system for integrating migrant children should not simply apply the logic of Bronfenbenner’s ecosystem (that the most influential forces are those of the microsystem or family, rather than the exosystem or community). Instead, it should emphasize the maintenance of children’s self-concept, creating child-friendly communities and welcoming school services, and improvements to the support systems and policies related to migrant children and their families’ socioeconomic status.

### 4.3. Study Limitations 

Social policy, cultural environment, and other macro systems are highly homogeneous for migrant children in Guangzhou and Foshan. As a result, this study only explored environmental systems (community, school, and family) that closely connect migrant children and can be recognized and perceived. There is, of course, the likelihood that social policies and other socio-cultural systems affect migrant children’s subjective social mobility. This research was also limited by its cross-sectional nature and the geographical restriction to Guangzhou and Foshan. With the acceleration of China’s urbanization, more migrant children will follow their parents to live, study, and work in cities. Migrant-children-oriented schooling, settlement, and support policies will change with time, and policy analysis, especially longitudinal policy studies, should be considered for subsequent in-depth research. Moreover, cities in China are highly diverse in managing migrant populations and accepting migrant children; thus, future policy studies should cover more locations. Globalization will generate an increase in migrant children from other countries with very different cultural backgrounds, and the impact of policies and cultural systems on their development also requires attention. After all, children need a hopeful world enabling them to feel “tomorrow will be a better day”.

## Figures and Tables

**Table 1:** Distributions of the target population and the study sample.

Variables	Distribution of Migrant Children Aged 8–14 in Guangdong	Distribution of the Study Sample
Age group:		
8–11 years	63.2%	61.2%
12–14 years	36.8%	38.8%
Gender:		
Male	57.4%	58.4%
Female	42.6%	41.6%

**Table 2:** Factor characteristics of the measurement model: reliability and convergence validity.

Reflective Factor					
Dimension	Items	Factor Loadings	*p* Value	Composite Reliability (CR)	Average Variance Extracted (AVE)
School integration ^1^	4	0.722–0.823	***	0.842	0.577
Emotional attachment to school	4	0.802–0.864	***	0.903	0.699
Teacher–student relationship	4	0.735–0.849	***	0.873	0.633
Classmate interaction	4	0.726–0.833	***	0.874	0.634
School performance	2	0.887–0.889	***	0.882	0.789
Community integration	5	0.691–0.791	***	0.858	0.548
Self-concept	6	0.712–0.866	***	0.908	0.624
Subjective class identification	1	1.000		1.000	1.000
Subjective social mobility	1	1.000		1.000	1.000
**Formative Factor**					
**Factor**	**Items**	**Weights**	***p* Value**	**Collinearity** **(VIF)**	
Family Socioeconomic Status	4	0.200–0.578	**	1.026–1.596	

Note: The *p* values displayed above are the highest among the items, ** = *p* < 0.01; *** = *p* < 0.001; ^1^. School integration is a second-order factor, while emotional attachment to school, teacher–student relationship, classmate interaction, and school performance are first-order factors. The two-stage factor score method was used to calculate the indicators of higher-order factors.

**Table 3:** Factor characteristics of the measurement model: discriminative validity.

Factor	Discriminative Validity									
	Family Socioeconomic Status	School Integration		Community Integration		Self-Concept		Subjective Class Identification		Class Mobility Expectation
Family socioeconomic status	-									
School integration	0.112	***0.760***								
Community integration	0.097	0.504	(0.650)	***0.740***						
Self-concept	0.147	0.587	(0.717)	0.499	(0.588)	***0.790***				
Subjective class Identification	0.148	0.107	(0.123)	0.134	(0.151)	0.151	(0.160)	***1.000***		
Subjective social Mobility	0.139	0.377	(0.437)	0.396	(0.443)	0.372	(0.390)	0.317	(0.317)	***1.000***

Note: The figures (bold and italic font) on the diagonal are the AVE square root values; the figures in the inferior triangle are the Pearson correlation coefficients; and the values in the “( )” are the HTMT values.

**Table 4:** Descriptive statistics.

Factor	Mean	*SD*	Median	Min	Max
Family socioeconomic status ^1^					
Father’s education level	1.892	1.081	1.000	1.000	4.000
Mother’s education level	1.764	1.025	1.000	1.000	4.000
Total monthly family income	1.990	0.884	2.000	1.000	4.000
Family living conditions	2.093	0.507	2.000	1.000	3.000
School integration	3.905	0.579	3.962	1.113	5.000
Classmate interaction	3.942	0.795	4.049	1.000	5.000
Teacher–student relationship	4.226	0.697	4.263	1.000	5.000
Emotional attachment to school	3.747	0.883	4.000	1.000	5.000
School performance	3.719	0.706	3.769	1.000	5.000
Community integration	3.779	0.780	3.825	1.000	5.000
Self-concept	0.681	0.163	0.693	0.074	0.990
Subjective class identification	3.869	2.421	3.000	1.000	10.000
Subjective social mobility	6.719	2.300	7.000	1.000	10.000

Note: *SD* = standard deviation. ^1^. Family socioeconomic status is a formative latent variable. The factor mean was not calculated, and only the basic statistical magnitudes (such as the means of specific items) were calculated.

**Table 5:** Structural model path coefficients.

Path Coefficient	Estimate	*SD*	T Value	*p* Value	Bootstrapping			
					Percentile		Bias-Corrected	
					2.5%	97.5%	2.5%	97.5%
Family socioeconomic status -> subjective social mobility	0.046	0.020	2.328	*	0.009	0.085	0.005	0.081
School integration -> subjective social mobility	0.165	0.025	6.730	***	0.117	0.213	0.120	0.217
Community integration -> subjective social mobility	0.213	0.023	9.224	***	0.167	0.258	0.168	0.258
Family socioeconomic status -> self-concept	0.063	0.017	3.704	***	0.032	0.099	0.029	0.096
School integration -> self-concept	0.442	0.020	21.907	***	0.402	0.481	0.402	0.481
Community integration -> self-concept	0.262	0.021	12.471	***	0.220	0.303	0.221	0.303
Self-concept -> subjective social mobility	0.125	0.023	5.311	***	0.079	0.170	0.078	0.169
Control variable								
Subjective class identification -> self-concept	0.059	0.017	3.491	***	0.025	0.091	0.026	0.091
Subjective class identification -> subjective social mobility	0.245	0.020	12.233	***	0.205	0.284	0.216	0.293

Note: * = *p* < 0.05; *** = *p* < 0.001.

**Table 6:** Mediating and total effects.

Self-Concept (a Mediating Variable)	Estimate	*SD*	T Value	*p* Value	Bootstrapping			
					Percentile		Bias-Corrected	
					2.5%	97.5%	2.5%	97.5%
**Indirect Effect**								
Family socioeconomic status -> subjective social mobility	0.008	0.003	3.071	**	0.004	0.014	0.003	0.014
School integration -> subjective social mobility	0.055	0.011	5.148	***	0.035	0.076	0.035	0.076
Community integration -> subjective social mobility	0.033	0.007	4.836	***	0.020	0.046	0.020	0.046
**Total Effect**								
Family socioeconomic status -> subjective social mobility	0.053	0.020	2.732	**	0.017	0.094	0.011	0.088
School integration -> subjective social mobility	0.220	0.022	9.926	***	0.177	0.264	0.178	0.266
Community integration -> subjective social mobility	0.246	0.022	11.032	***	0.202	0.289	0.203	0.289

Note: ** = *p* < 0.01; *** = *p* < 0.001.

**Table 7:** Influence, performance, and importance.

	Subjective Social Mobility		
	Influence (*ƒ*^2^)	Performance	Importance
Family socioeconomic status	0.003	42.570	0.265
School integration	0.022	72.632	0.875
Community integration	0.043	69.486	0.725
Self-concept	0.013	68.032	1.754
Subjective class identification	0.080	31.883	0.240
*R* ^2^	0.277		
*Q* ^2^	0.271		

**Table 8:** Permutation group tests.

Factor	Boy–Girl						Primary School–Secondary School					
	Difference of Factor Loading/Weight ^1^		Difference of Factor Mean		Difference of Factor Variance		Difference of Factor Loading/Weight		Difference of Factor Mean		Difference of Factor Variance	
	2.5%	97.5%	2.5%	97.5%	2.5%	97.5%	2.5%	97.5%	2.5%	97.5%	2.5%	97.5%
Family socioeconomic status	−0.342	0.371	−0.083	0.082	−0.125	0.136	−0.323	0.380	−0.093	0.084	−0.139	0.139
School integration	−0.036	0.035	−0.086	0.089	−0.137	0.145	−0.037	0.037	−0.087	0.089	−0.149	0.139
Community integration	−0.041	0.039	−0.082	0.088	−0.128	0.130	−0.041	0.043	−0.086	0.086	−0.125	0.131
Self-concept	−0.020	0.019	−0.082	0.082	−0.111	0.117	−0.021	0.018	−0.087	0.083	−0.113	0.120
Subjective class Identification	-	-	−0.080	0.082	−0.122	0.119	-	-	−0.083	0.087	−0.120	0.103
Subjective social Mobility	-	-	−0.083	0.083	−0.104	0.109	-	-	−0.084	0.079	−0.107	0.110

Note: ^1^. Each factor contains several measurement indexes. In order to reduce the table length, only the absolute differences between the 2.5% and 97.7% percentiles of the measurement indexes that are closest to “0” are listed. Factor weight only applies to family socioeconomic status factor, and factor loading applies to other factors.

**Table 9:** Comparison of path coefficients and intermediary effects of subgroups.

Path Coefficient	Boys–Girls		Permutation		Primary School–Secondary School		Permutation	
	Original Difference	Permutated Average Difference	2.5%	97.5%	Original Difference	Permutated Average Difference	2.5%	97.5%
Family socioeconomic status -> self-concept	0.057	−0.002	−0.071	0.064	0.020	0.000	−0.069	0.063
Family socioeconomic status -> subjective social mobility	−0.001	−0.003	−0.082	0.075	0.013	−0.003	−0.076	0.074
School integration -> self-concept	−0.034	−0.003	−0.091	0.082	0.183	−0.004	−0.085	0.078
School integration -> subjective social mobility	0.038	0.000	−0.096	0.099	0.022	0.002	−0.097	0.103
Community integration -> self-concept	0.004	0.002	−0.086	0.093	0.036	0.001	−0.051	0.054
Community integration -> subjective social mobility	−0.015	0.001	−0.087	0.094	−0.075	0.000	−0.041	0.045
Self-concept -> subjective social mobility	0.012	−0.002	−0.094	0.091	0.061	−0.001	−0.056	0.056
Subjective class identification -> self-concept	0.037	0.002	−0.061	0.067	−0.021	0.000	−0.036	0.036
Subjective class identification -> subjective social mobility	−0.004	0.001	−0.077	0.080	−0.026	0.002	−0.076	0.084
**Self-concept (mediating effect)**								
Family socioeconomic status -> subjective social mobility	0.008	0.000	−0.011	0.010	0.005	0.000	−0.012	0.010
School integration -> subjective social mobility	0.001	−0.001	−0.041	0.041	0.040	−0.001	−0.045	0.039
Community integration -> subjective social mobility	0.004	0.000	−0.026	0.025	0.010	0.000	−0.027	0.026

## Data Availability

The raw data supporting the conclusions of this article will be made available by the authors without undue reservation.
